# Everybody's Page

**Published:** 1890-04-05

**Authors:** 


### Everybody's Page.

Starfish are the greatest enemies of the oyster beds.

The dolphin can swim at the rate of twenty miles an hour, but the Spanish mackerel can beat him. The swiftest swimmers of all are the marine highwaymen—the sharks and the dog-fishes. Perhaps this is the result of long practice, and much chasing of prey.

A leaf which we might take out of the French cookery book is their use of many sorts of salad. In England we nearly always have lettuce or endive or beetroot, but in France they grow and eat many more varieties of salad than we do, such as common chicory, dandelion blanched and half-blanched, salsify tops, witloof, purslane (which was formerly much grown in English gardens), rampion, nasturtium flowers, and many others.

The formula formerly used in France when the King touched scrofulous people was "The King touches thee; may God cure thee," but it seems that it was not only Kings who had this marvellous healing power. A "Marcou" was also able to effect a cure. A Marcou is the seventh son of a man, born in direct male succession, and when he is born he is supposed to have a birth-mark of the Fleur-de-Lis somewhere on his body, and if he only breathes on a scrofulous person the effect is miraculous. The office for the ceremony of touching for King's Evil remained in our liturgy as late as 1719.

Nature in her bounty seems to provide a substitute for all exhausted products if mankind will only exert themselves to search and experimentalise. Numerous chemical agencies have of late years been used in the production of leather in the United States, owing to the growing scarcity of oak-bark and other suitable vegetable products. The Australian wattle, which is entensively cultivated in the Colonies of New South Wales and Victoria, is now coming to the rescue. Of two varieties, the black and the broad-leaved, the former produces the larger amount of tannic acid in the bark. The wood of both these varieties is of use for various purposes.

"Sunlight and fresh air are essential to healthy life, and an adequate supply of both the basis of all hygiene," to quote from Dr. J. Edward Squire's paper on the "Prevention of Consumption." While to the general public the word small-pox acts as a scare, phthisis is barely understood; yet this disease carries off annually "more than a fourth of all the males who die during the working period of life." Dr. Squire tells us how much influence "Sunlight and fresh air" have on the ravages of phthisis; he describes the classes who suffer from dusty employments, owing to the inhalation of irritating particles of dust, which impair the lungs, and how much can be done for the workers by efficient ventilation and cleanliness. Then there are the home workers (whom it is almost an impossibility to bring under sanitary control), who sit and stitch, perhaps six or seven souls all stuffed into one miserable room with a window which won't open; a veritable hot-bed for the tubercle bacillus. Dr. Squire suggests public work-rooms, some where men could for a very small sum take their work to do, and others where seamstresses and waistcoat makers might stitch in comfort, and the one wretched room might then be a little more like a home to go to when the work was done. But the worst of it is that the poor people don't appreciate fresh air altogether; perhaps they will in time; meanwhile, what more charitable lesson can the philanthropist, in the true meaning of the word, teach, than the simple rudiments of hygiene?

A common country cure for weak eyes is a poultice of "rotten apples."

The Government Telegraph service of Great Britain transmits, it is said, on the average 1,538,270 words a-day to newspapers alone.

The votaries of a certain prevalent fashion deserve to be martyrs of neuralgia, if they are not. We notice ladies wrapped up in furs as if they were starting for the North Pole, and on their heads a bonnet made literally of a bunch of violets or two pansies. Spring like, no doubt, these bonnets and inexpensive, but when will people believe that exaggerated fashion is devoid of either use or beauty?

The Japanese Emperor will have no more of duelling, and the new legislation against it is such that it would take a brave Jap now to challenge a man to mortal combat. Fancy being detected in merely sending a challenge to a foe for some dire offence, and being whisked off to prison and hard labour without accomplishing one dig at the offender. And this is only one of the minor punishments. Whoever shall have engaged in a duel shall be imprisoned with hard labour from two to five years. Such drastic measures ought to quiet the most pugilistically inclined.

Mr. Thomas Tipple was a gentleman of renown (medical renown at least), for, in the year 1812, he was taking a horse out of a chaise, when the animal plunged forward, and the shaft penetrated the chest of the poor man, going in under his left arm and coming out under the right arm, pinning him to the door of the coach-house. But this was not the end of Mr. Tipple. He was treated by Mr. William Maiden, and whether he recovered in spite of or on account of the treatment, it is hard to say; but he did recover. A pamphlet written at the time states that he was well bled—very well, if we may believe that in ten days he lost 184 ounces of blood—and was given blisters, and calomel, and Epsom salts as well, and was sustained by a vegetable diet! Mr. Tipple lived eleven years after the accident, and became Master of Hoxton Workhouse. Who shall say that, after all this, he was not entitled to renown? The Royal College of Surgeons evidently thought he was, for they preserved the shaft in their museum.

We all grumble when infectious illness compels us to pull our houses to bits and send everything off to be disinfected, but a contributor to last week's *Journal d'Hygiene* gives a few of the methods of disinfection in the seventeenth century mentioned in some curious old document, and they were evidently no more conducive to comfort than those of the present day. After a violent epidemic of plague in Montpellier all the inhabitants had to leave their houses empty and depart out of the town to allow them to be fumigated. Aromatic plants—thyme, lavender, etc.—were burnt, and all clothes and bedding exposed to their fumes. The house was swept with brooms of furze, the walls washed down with lie-wash; then the doors and windows were flung open, and the perfumers arrived and fumigated with hay sprinkled with wine and vinegar. The third day antiseptics, such as sulphur, mercury, antimony, were used; and the fourth day the strong smell of these latter was driven out by sweet-smelling incense and myrrh. Anything which could prove a source of danger was burnt, all dust and dirt carted off far away, and the streets received an immense amount of attention in the way of sweeping and scraping. What the poor inhabitants did with themselves during these elaborate proceedings we are not told.

